# Echocardiographic Assessment of Biventricular Mechanics in Patients with Mild-to-Moderate Idiopathic Pulmonary Fibrosis: A Systematic Review and Meta-Analysis

**DOI:** 10.3390/jcm14030714

**Published:** 2025-01-22

**Authors:** Andrea Sonaglioni, Antonella Caminati, Gian Luigi Nicolosi, Giovanna Elsa Ute Muti-Schünemann, Michele Lombardo, Sergio Harari

**Affiliations:** 1 Division of Cardiology, IRCCS MultiMedica, 20123 Milan, Italy; michele.lombardo@multimedica.it; 2 Semi-Intensive Care Unit, Division of Pneumology, IRCCS MultiMedica, 20123 Milan, Italy; antonella.caminati@multimedica.it (A.C.); sergio.harari@unimi.it (S.H.); 3 Division of Cardiology, Policlinico San Giorgio, 33170 Pordenone, Italy; gianluigi.nicolosi@gmail.com; 4 Department of Emergency, Fondazione IRCSS Ca’ Granda, Ospedale Maggiore Policlinico, 20122 Milan, Italy; giovanna.muti@unimi.it; 5 Department of Clinical Sciences and Community Health, Università di Milano, 20122 Milan, Italy

**Keywords:** idiopathic pulmonary fibrosis, subclinical myocardial dysfunction, speckle tracking echocardiography, myocardial strain, biventricular mechanics

## Abstract

**Background:** Over the last few years, a few imaging studies have performed conventional transthoracic echocardiography (TTE) implemented with speckle tracking echocardiography (STE) for the assessment of biventricular mechanics in patients with non-advanced idiopathic pulmonary fibrosis (IPF). This systematic review and meta-analysis aimed at evaluating the overall effect of mild-to-moderate IPF on the main indices of biventricular systolic function assessed by TTE and STE. **Methods:** All imaging studies assessing right ventricular (RV)-global longitudinal strain (GLS), left ventricular (LV)-GLS, tricuspid annular plane systolic excursion (TAPSE), and left ventricular ejection fraction (LVEF) in IPF patients vs. healthy controls, selected from PubMed, Scopus, and EMBASE databases, were included. Continuous data (RV-GLS, LV-GLS, TAPSE, and LVEF) were pooled as standardized mean differences (SMDs) comparing the IPF group with healthy controls. The SMD of RV-GLS was calculated using the random-effect model, whereas the SMDs of LV-GLS, TAPSE, and LVEF were calculated using the fixed-effect model. **Results:** The full texts of 6 studies with 255 IPF patients and 195 healthy controls were analyzed. Despite preserved TAPSE and LVEF, both RV-GLS and LV-GLS were significantly, although modestly, reduced in the IPF patients vs. the controls. The SMD was large (−1.01, 95% CI −1.47, −0.54, *p* < 0.001) for RV-GLS, medium (−0.62, 95% CI −0.82, −0.42, *p* < 0.001) for LV-GLS, small (−0.42, 95% CI −0.61, −0.23, *p* < 0.001) for TAPSE, and small and not statistically significant (−0.20, 95% CI −0.42, 0.03, *p* = 0.09) for LVEF assessment. Between-study heterogeneity was high for the studies assessing RV-GLS (I^2^ = 80.5%), low-to-moderate for those evaluating LV-GLS (I^2^ = 41.7%), and low for those measuring TAPSE (I^2^ = 16.4%) and LVEF (I^2^ = 7.63%). The Egger’s test yielded a *p*-value of 0.60, 0.11, 0.31, and 0.68 for the RV-GLS, LV-GLS, TAPSE, and LVEF assessment, respectively, indicating no publication bias. On meta-regression analysis, none of the moderators was significantly associated with effect modification for RV-GLS (all *p* > 0.05). The sensitivity analysis supported the robustness of the results. **Conclusions:** RV-GLS impairment is an early marker of subclinical myocardial dysfunction in mild-to-moderate IPF. STE should be considered for implementation in clinical practice for early detection of RV dysfunction in IPF patients without advanced lung disease.

## 1. Introduction

Idiopathic pulmonary fibrosis (IPF) is the most common form of idiopathic interstitial pneumonia, with rapid progression and a mean survival from diagnosis of 3 to 5 years [1]. IPF patients have a high burden of cardiovascular disease [2]. The principal cardiovascular complications of IPF include pulmonary hypertension (PH), right heart failure (RHF), coronary artery disease, carotid atherosclerosis, and cardiac arrhythmias [3,4,5,6]. Given that the cardiovascular complications have a negative impact on the prognosis and outcome of IPF patients [7], the identification of an innovative biomarker of early cardiovascular disease, that could independently predict the occurrence of adverse cardiovascular events, might improve the prognostic risk stratification of these patients.

Two-dimensional (2D) transthoracic echocardiography (TTE) is the most widely used imaging modality for the cardiovascular evaluation of IPF patients. It allows a simple, quick, and non-invasive assessment of the cardiac chamber cavity size, biventricular systolic function, and pulmonary hemodynamics. However, due to a number of intrinsic limitations of TTE, a relevant discordance between TTE and right heart catheterization in estimating pulmonary artery pressures is increasingly recognized [8,9,10]. Indeed, TTE can frequently overestimate or underestimate pulmonary artery pressure in PH patients. Moreover, TTE may provide limited information concerning cardiac morphology and function during the early stages of IPF.

Recent technological advances have led to the development of speckle tracking echocardiography (STE), a new imaging modality that accurately evaluates the myocardial deformation properties of both ventricles and atria. Differently from TTE, STE is able to detect the subclinical myocardial dysfunction, defined as the reduction in right ventricular (RV) and/or left ventricular (LV)-global longitudinal strain (GLS), in the presence of preserved tricuspid annular plane systolic excursion (TAPSE) and/or left ventricular ejection fraction (LVEF) [11,12].

To date, myocardial strain parameters have been poorly investigated in IPF patients. Over the last decade, only a few imaging studies have performed conventional TTE implemented with strain echocardiographic imaging for the assessment of biventricular mechanics in IPF patients without advanced lung disease. These studies aimed at identifying an innovative prognostic indicator of early subclinical myocardial dysfunction in patients with early-stage IPF. Accordingly, we performed a systematic review and meta-analysis to summarize the main findings of these echocardiographic studies and to assess the overall effect of mild-to-moderate IPF on the main conventional and innovative indices of biventricular systolic function.

## 2. Materials and Methods

This systematic review and meta-analysis was conducted according to the PRISMA guidelines [13] and was registered in the PROSPERO database (CRD42024577785).

### 2.1. Search Strategy

A comprehensive search of all imaging studies assessing conventional echocardiographic indices and myocardial strain parameters in patients with mild-to-moderate IPF vs. healthy controls without IPF was carried out by two independent reviewers (A.S. and M.L.) through August 2024, by using the PubMed, Scopus, and EMBASE databases. The search strategy included the following terms: “idiopathic pulmonary fibrosis (IPF)” OR “fibrosing interstitial lung disease (F-ILD)” AND “biventricular systolic function” OR “tricuspid annular plane systolic excursion (TAPSE)” OR “left ventricular ejection fraction (LVEF)” AND “biventricular myocardial strain” OR “right ventricular global longitudinal strain (RV-GLS)” OR “left ventricular global longitudinal strain (LV-GLS).” The search was extended to include full-text articles published in all languages. There was no limitation of time period.

### 2.2. Eligibility Criteria

All echocardiographic studies evaluating conventional and innovative indices of biventricular systolic function in IPF patients without severe PH vs. controls without IPF matched by age, sex, and cardiovascular risk factors were included. Only studies performed on patients with early-stage and/or mild-to-moderate IPF, defined by forced vital capacity (FVC) > 50%, diffusing capacity of the lungs for carbon monoxide (DLCO) > 35%, TTE-derived tricuspid regurgitation velocity (TRV) < 3.4 m/sec [14], Gender-Age-Physiology Index score ≤ 5 [15], and modified Medical Research Council scale score ≤3 [16] were included. Conversely, imaging studies conducted on IPF patients with advanced lung disease, defined by FVC < 50%, DLCO < 35%, and severe PH (TRV ≥ 3.4 m/sec) [14] and/or congestive RHF; imaging studies performed on IPF patients without controls; imaging studies with incomplete echocardiographic data; imaging studies that did not analyze myocardial strain parameters in IPF patients; non-clinical articles; animal studies; duplicate articles; case reports; reviews; editorials; research letters without data; and abstracts were excluded.

### 2.3. Study Selection and Data Extraction

Two reviewers (A.S. and M.L.) screened the databases according to the inclusion criteria and performed data extraction independently. Information concerning the following was independently collected by the two reviewers: (1) demographics (age and sex); (2) anthropometrics [body mass index (BMI)]; (3) prevalence of relevant cardiovascular risk factors (smoking, hypertension, and type 2 diabetes mellitus); (4) relevant comorbidities, such as history of coronary artery disease (CAD); (5) hemodynamics (heart rate, systolic and diastolic blood pressure); (6) pulmonary function tests (PFTs), DLCO and six-minute walking test (6 MWT); (7) conventional indices of cardiac morphology and function assessed by TTE; (8) RV and LV myocardial strain parameters assessed by STE; (9) the current medical treatment; and (10) follow-up data (if any). Possible discrepancies between the reviewers were resolved through a consensus discussion with the involvement of a third author (G.L.N.).

### 2.4. Risk of Bias Assessment

The articles included in this systematic review and meta-analysis were assessed for risk of bias (RoB) using the National Institutes of Health (NIH) Quality Assessment of Case-Control Studies [17]. The quality rating was independently estimated by two authors (A.S. and G.L.N.). Disagreement was resolved by consensus.

**Figure 1:**
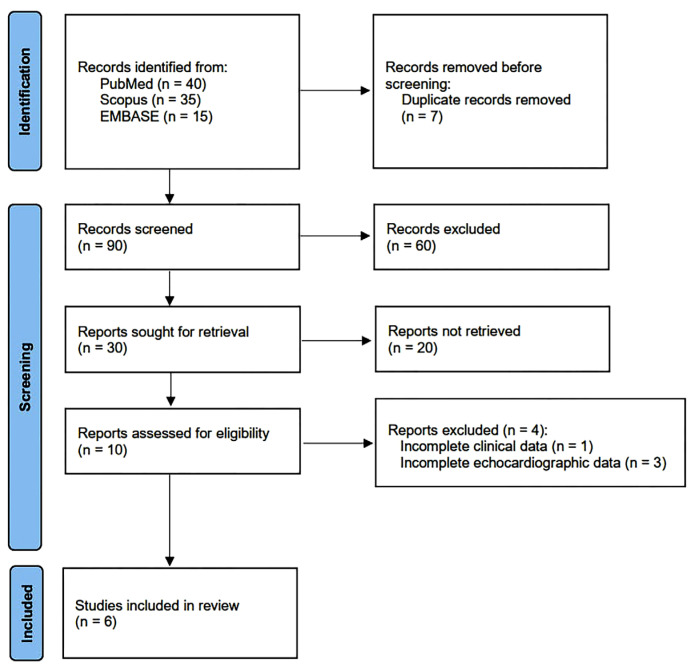
Flow diagram used for identifying the included studies.

The PRISMA flow diagram used for identifying the included studies is depicted in Figure 1.

### 2.5. Statistical Analysis

The primary endpoint was to investigate the effect of IPF on both innovative (RV-GLS and LV-GLS) and traditional (TAPSE and LVEF) indices of biventricular mechanics. For the purpose of the study, RV-GLS, LV-GLS, TAPSE, and LVEF were expressed as means with deviations. Continuous data (RV-GLS, LV-GLS, TAPSE, and LVEF) were pooled as standardized mean differences (SMDs) comparing the IPF group with the controls. The SMD of RV-GLS was calculated using the random-effect model, due to the high statistical heterogeneity among the included studies, with regard to study design, sample size, demographics, spirometry parameters, and the specific imaging used for the assessment of RV mechanics in IPF patients. On the other hand, the SMDs of LV-GLS, TAPSE, and LVEF were calculated using the fixed-effect model, due to a low between-study heterogeneity in assessing these parameters. The I-squared statistic (I^2^) was used to quantify the degree of statistical heterogeneity among the studies. Begg’s funnel plots and the Egger’s test were employed to assess potential publication bias for RV-GLS, LV-GLS, TAPSE, and LVEF measurement. Meta-regression was performed to evaluate the effect modification on RV-GLS by the following moderators: age, hypertension, type 2 diabetes, and smoking. The 95% confidence intervals (CIs) was calculated, and two-tailed *p* values < 0.05 were considered statistically significant. Finally, a sensitivity analysis was performed by investigating the effect of individual studies on the overall meta-analysis: The meta-analysis was re-estimated by omitting each study, sequentially, to determine the robustness of the results. The statistical analysis was performed by using Comprehensive Meta-Analysis version 3.0 (Biostat, Englewood, NJ, USA).

## 3. Results

### 3.1. Clinical Findings

The initial search yielded a total of 97 studies. Of those, 7 (7.2%) were removed as duplicates. After screening the titles and abstracts, a further 80 studies (82.5%) were removed on the basis of the exclusion criteria. The evaluation of the full text of the remaining 10 studies (10.3%) resulted in further 4 exclusions (4.1%). A total of 6 studies (6.2%) [18,19,20,21,22,23] were thus included in this systematic review and meta-analysis, totalling 255 IPF patients and 195 healthy controls without IPF.

The clinical characteristics and the main findings of the included studies are reported in Table 1.

The included studies were published between 2016 and 2022. Five studies were performed in Italy, one in Brazil. All the included studies were monocentric. Four studies (66.7% of the total) had a prospective design, whereas the remaining two (33.3% of the total) had a retrospective design. The mean age of the IPF patients among the included studies was 68.5 yrs (range 61.2–73.8 yrs). Approximately two-third (66% of total) of the IPF patients were males. All the included studies performed resting 2D-TTE implemented with STE analysis of biventricular mechanics. The majority of the studies (66.7% of total) examined biventricular myocardial strain by using General Electric (GE) software, one study used Esaote software, and one study used Philips software. After adequate tracking of the RV and LV myocardial walls, each software automatically divided both the RV and LV contours into six segments. RV-GLS was uniformly calculated from the apical four-chamber view by averaging local strains obtained from three septal and three lateral segments, whereas LV-GLS was measured as the average of the individual peak strain derived from six segments in each of the three apical views. Differently from the other authors, Buonauro A. et al. [20] performed a three-dimensional (3D) echocardiographic examination of RV size and function. Interestingly, D’Andrea A. et al. [19] and Cobra S.B. et al., (2021) [22] evaluated biventricular systolic function both at rest and during exercise stress testings.

Table 2 reports all the clinical, respiratory, and echocardiographic parameters obtained in the IPF patients and controls by the included studies.

The IPF patients were predominantly males, aged >65 yrs, with a moderate prevalence of smoking and hypertension and a low prevalence of type 2 diabetes. Information regarding comorbidities was provided by only two studies [21,23], demonstrating a low-to-moderate prevalence of CAD history.

### 3.2. Instrumental Findings

PFTs revealed a mild impairment in average FEV1%, a mild-to-moderate impairment in average FVC%, and a moderate reduction in average DLCO% in the IPF patients. The 6 min walking distance covered by the IPF patients was slightly reduced in comparison to the accepted normal values for healthy elderly individuals [24]. Compared to the controls, the IPF patients had significantly higher heart rate and higher systolic and diastolic blood pressure values.

On conventional 2D-TTE, the IPF patients and the controls were found with similar left-sided cardiac chamber cavity size and similar LV systolic function, as assessed by LVEF. Analysis of LV diastolic function revealed that the transmitral E/A ratio was significantly lower and the E/e’ ratio was significantly higher in the IPF patients than the controls, thus indicating a first degree of diastolic dysfunction associated with a mild increase in LV filling pressures in patients with mild-to-moderate IPF. As expected, the IPF patients were diagnosed with larger RV basal diameter and higher pulmonary artery pressures than the controls. However, the average TRV value obtained in the IPF patients (2.66 m/sec) did not reach the echocardiographic criteria for diagnosing PH [14].

Despite the preserved traditional indices of biventricular systolic function (TAPSE and LVEF) on conventional 2D-TTE, STE analysis showed that all the main parameters of RV and LV myocardial deformation were significantly, although modestly, reduced in the IPF patients in comparison to the controls and to the accepted reference values [12,25]. A concomitant attenuation of left atrial reservoir strain (LASr) in the IPF patients was demonstrated by our study group.

**Figure 2:**
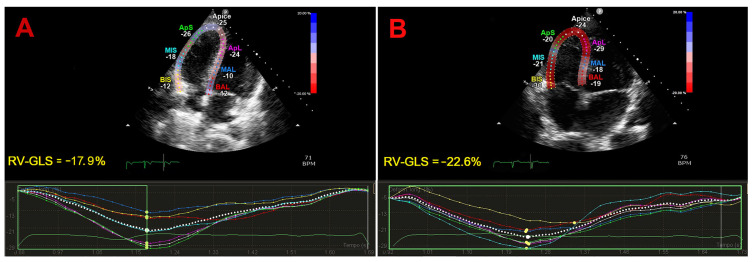
Representative examples of RV-GLS magnitude obtained by STE in a patient with mild-to-moderate IPF (**A**) and in a healthy control without IPF (**B**). The dotted line indicates the average RV-GLS, determined by averaging strain values of the segments from the free lateral wall and the segments from the interventricular septal wall. GLS, global longitudinal strain; IPF, idiopathic pulmonary fibrosis; RV, right ventricular; STE, speckle tracking echocardiography.

Representative examples of STE-derived RV-GLS obtained in a patient with mild-to-moderate IPF and in a healthy individual without IPF are illustrated in Figure 2, Panels A and B, respectively.

### 3.3. Risk-of-Bias Assessment

Regarding the RoB, the NIH quality rating was estimated as fair for five studies and good for one study. The Cohen’s Kappa coefficient for the agreement between the reviewers in the RoB assessment indicated excellent agreement, κ = 0.85.

### 3.4. Effect of IPF on RV-GLS

**Figure 3:**
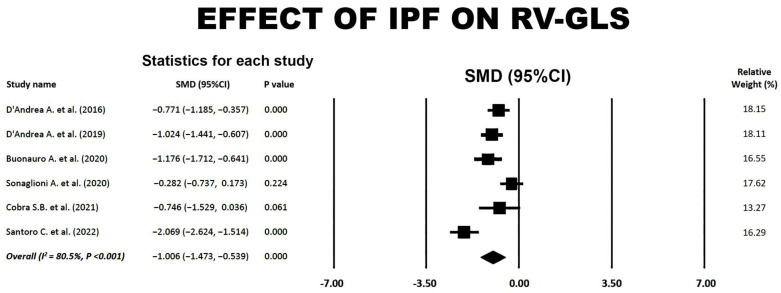
Forest plot showing the effect of IPF on RV-GLS in the included studies [18,19,20,21,22,23]. CI, confidence interval; GLS, global longitudinal strain; IPF, idiopathic pulmonary fibrosis; RV, right ventricular; SMD, standardized mean difference.

Forest plot illustrating the effect of IPF on RV-GLS is depicted in Figure 3.

**Figure 4:**
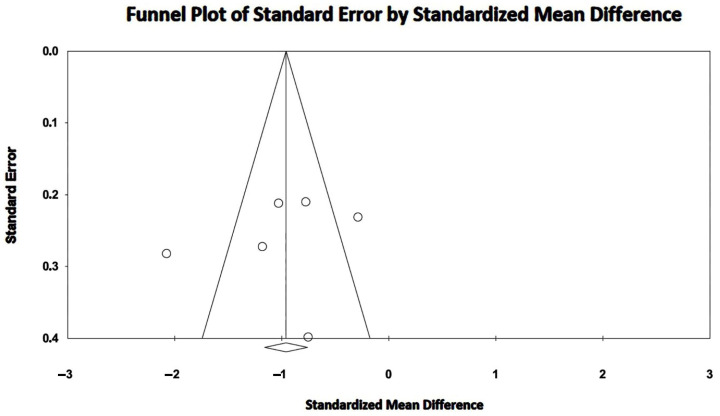
Begg’s funnel plot for the detection of publication bias with regard to RV-GLS assessment by the included studies. GLS, global longitudinal strain; RV, right ventricular.

Large SMDs were obtained for the included studies, with an overall SMD of −1.01 (95% CI −1.47, −0.54, *p* < 0.001). Substantial heterogeneity was detected among the included studies, with an overall I^2^ statistic value of 80.5% (*p* < 0.001). The Egger’s test for a regression intercept yielded a *p*-value of 0.60, indicating no publication bias. The Begg’s funnel plot for the detection of publication bias is illustrated in Figure 4.

On meta-regression analysis, none of the moderators (age, hypertension, type 2 diabetes, smoking) was significantly associated with effect modification on the association between IPF and RV-GLS (all *p* > 0.05) (Table 3).

The sensitivity analysis supported the robustness of the results. Omitting each study sequentially caused a modest variability in SMD, from −0.96 (95% CI −1.28, −0.64) to −2.07 (95% CI −2.62, −1.51).

### 3.5. Effect of IPF on LV-GLS

**Figure 5:**
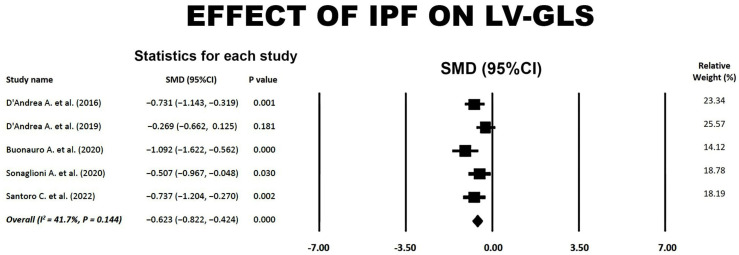
Forest plot showing the effect of IPF on LV-GLS in the included studies [18,19,20,21,23]. CI, confidence interval; GLS, global longitudinal strain; IPF, idiopathic pulmonary fibrosis; LV, left ventricular; SMD, standardized mean difference.

Forest plot showing the effect of IPF on LV-GLS is illustrated in Figure 5.

The overall SMD of −0.62 (95% CI −0.82, −0.42, *p* < 0.001) indicated a medium effect of IPF on LV-GLS. A low-to-moderate between-study heterogeneity was obtained, with an overall I^2^ statistic value of 41.7% (*p* = 0.144). The Egger’s regression intercept gave a *p*-value of 0.11, indicating no publication bias.

### 3.6. Effect of IPF on TAPSE

**Figure 6:**
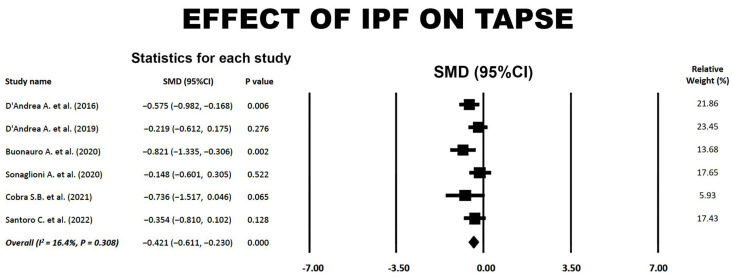
Forest plot showing the effect of IPF on TAPSE in the included studies [18,19,20,21,22,23]. CI, confidence interval; IPF, idiopathic pulmonary fibrosis; SMD, standardized mean difference; TAPSE, tricuspid annular plane systolic excursion.

A forest plot illustrating the effect of IPF on TAPSE is reported in Figure 6.

### 3.7. Effect of IPF on LVEF

**Figure 7:**
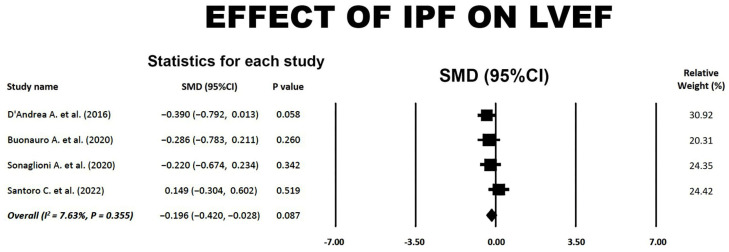
Forest plot showing the effect of IPF on LVEF in the included studies [18,20,21,23]. CI, confidence interval; IPF, idiopathic pulmonary fibrosis; LVEF, left ventricular ejection fraction; SMD, standardized mean difference.

A forest plot summarizing the effect of IPF on LVEF is shown in Figure 7.

The overall SMD of −0.20 (95% CI −0.42, 0.03, *p* = 0.09) indicated a small and not statistically significant effect of IPF on LVEF. A low between-study heterogeneity was detected, with an overall I^2^ statistic value of 7.63% (*p* = 0.355). The Egger’s regression intercept gave a *p*-value of 0.68, indicating no publication bias.

## 4. Discussion

### 4.1. Main Findings of the Present Systematic Review and Meta-Analysis

This systematic review and meta-analysis, which analyzed echocardiographic studies performed on IPF patients without advanced lung disease, demonstrated that, compared to the matched controls, the IPF patients had the following: (1) significantly higher heart rate and blood pressure values; (2) similar left-sided cardiac chamber internal dimensions and LV systolic function; (3) significantly higher LV filling pressures; (4) significantly larger RV basal diameter, lower TAPSE, and higher sPAP; (5) significantly lower biventricular myocardial strain parameters. However, all the above-mentioned hemodynamic and echocardiographic changes were subtle, falling within the accepted reference ranges. Overall, the echocardiographic phenotype associated with mild-to-moderate IPF was characterized by normal cardiac chamber cavity sizes, normal LVEF, first degree of diastolic dysfunction, E/e’ ratio in the gray zone between 8 and 13, mild RV dilatation, normal TAPSE, and mild attenuation of both RV-GLS and LV-GLS.

The meta-analysis results revealed that the overall effect of IPF on biventricular mechanics was large for RV-GLS, medium for LV-GLS, and small for both TAPSE and LVEF. The effect of IPF on RV-GLS was not influenced by potential confounders, such as age, hypertension, type 2 diabetes, and smoking. The high between-study heterogeneity for RV-GLS assessment, detected by the I^2^ statistic, was likely related to the different types of software used for the STE analysis, to the not univocal study design, and to different patient characteristics with regards to age, gender, cardiovascular risk factors distribution, and IPF degree (mild vs. moderate).

### 4.2. Pathophysiological Mechanisms Underpinning Myocardial Strain Impairment in Mild-to-Moderate IPF

Due to the load-dependence of peak systolic strain [26], the early RV-GLS impairment detected in non-advanced IPF has been related to subclinical levels of elevated mean pulmonary artery pressure (mPAP) [14]. Similar findings have already been documented in chronic obstructive pulmonary disease (COPD) patients with a slight increase in mPAP [27]. RV-GLS enables detection of subclinical PH even when conventional indices of RV systolic function are in the normal range. Differently from TAPSE, RV-GLS is angle-independent, less load-dependent, highly reproducible, and provides an accurate assessment of regional myocardial deformation [28].

During the early stage of IPF, additive mechanisms such as systemic inflammation and endothelial dysfunction might have a negative impact on the RV myocardial function [29].

Recent evidence also suggests that the fibrotic process which affects IPF patients is not limited to the lung parenchyma but may be extended to the arterial walls [30]. Poor pulmonary function and a low grade of systemic inflammation may synergically trigger the process of fibrotic remodeling within the vascular walls of arteries, thus contributing to cause vascular changes and increasing the aortic stiffness [31,32]. Due to its thin walls, the RV may be particularly susceptible to the increased systemic afterload, with consequent early subclinical RV-GLS deterioration.

Even if our meta-regression analysis excluded the potential influence of relevant cardiovascular risk factors on myocardial strain parameters, it is likely that the combined action of age, hypertension, and previous history of smoking may initiate and sustain a myopathic process involving not only the RV but also the left-sided cardiac chambers.

It is also possible that the undertreatment with cardio-protective drugs, such as beta-blockers, antihypertensives and statins, frequently observed in IPF patients [5,7], might contribute to accelerate the subclinical myocardial dysfunction detected in IPF patients.

**Figure 8:**
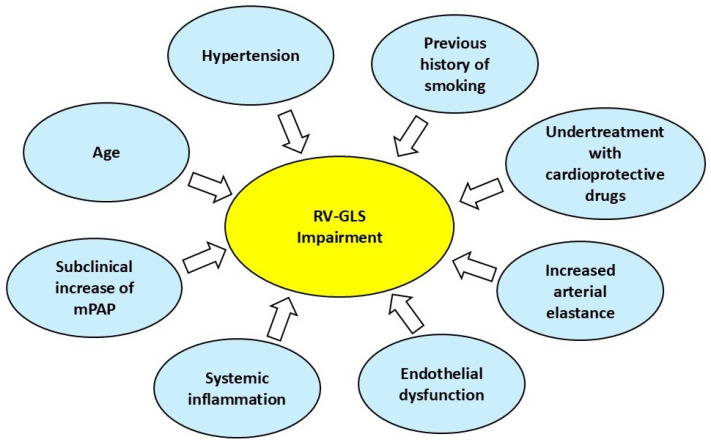
Relevant pathophysiological mechanisms underpinning RV-GLS impairment in non-advanced IPF. GLS, global longitudinal strain; IPF, idiopathic pulmonary fibrosis; mPAP, mean pulmonary artery pressure; RV, right ventricular.

All the aforementioned pathophysiological mechanisms underpinning RV-GLS impairment in non-advanced IPF are summarized in Figure 8.

Considering that the right ventricle and the left ventricle have common myocardial fibers, share the interventricular septum, and are wrapped within the pericardium [33], the RV dysfunction, typical of early stages of IPF, can also influence LV myocardial diastolic properties. LV diastolic dysfunction, impaired LV filling, and LV-GLS attenuation may occur as a consequence of septal wall distortion towards the left ventricle due to RV pressure overload [34,35,36]. In this clinical setting, the LASr impairment may be secondary to LV diastolic dysfunction [21]. Additionally, the subclinical impairment of both LV-GLS and LASr may be related to asymptomatic CAD and/or coronary artery calcifications that may be detected in a percentage of IPF patients ranging between 12% and 26% [37].

IPF patients at non-advanced stages may suffer from significantly decreased exercise tolerance due to RV-pulmonary artery (PA) uncoupling [19,22]. This uncoupling results from the increasing pressure workload represented by the “sick pulmonary circulation” and the inability of the right ventricle to adapt its contractile function to the increased metabolic demand. Indeed, the thinner RV wall is inadequate to preserve RV systolic function against increased pulmonary artery pressures during physical exercise [38,39]. The RV myocardial dysfunction, in its turn, is responsible for the insufficient increase in pulmonary blood flow, with further impairment of pulmonary gas exchange that finally results in a reduced tolerance to exercise [19,22].

### 4.3. Implications for Clinical Practice

Given the incremental diagnostic and prognostic value of STE over conventional TTE, strain echocardiographic imaging should be considered for implementation in the clinical practice for a more comprehensive evaluation of IPF patients without severe PH. Indeed, STE analysis may reveal a subclinical myocardial dysfunction characterized by the early deterioration of biventricular strain parameters, especially RV-GLS, in the presence of normal cardiac chamber cavity sizes and normal conventional indices of biventricular systolic function (TAPSE and LVEF), and in the absence of echocardiographic signs of PH. In addition, exercise stress echocardiography (ESE) or cardiopulmonary exercise testing (CPET) in combination with STE could be efficient screening tools to slatentize and/or amplify a resting subtle RV dysfunction in non-advanced IPF patients [19,22].

From a clinical point of view, IPF patients with mild-to-moderate lung disease might undergo TTE implemented with 2D-STE analysis of biventricular mechanics at basal evaluation and every six months, in order to recognize early cardiac dysfunction. ESE or CPET with simultaneous STE for evaluating biventricular mechanics both at rest and at peak exercise might allow the clinicians to obtain incremental diagnostic and prognostic information in IPF patients who are in an early disease stage. Among IPF patients, those with RV- and/or LV-GLS attenuation at rest and/or at peak exercise might need closer follow-up.

The assessment of myocardial strain parameters is particularly indicated for IPF patients with mild-to-moderate disease stage, in the absence of right-sided chamber dilatation and/or echocardiographic signs of chronic cor pulmonale. Even in the absence of severe pulmonary hypertension, a TTE-derived RV/LV basal diameter ratio ≥1 might be diagnostic of an advanced myocardial dysfunction, thus indicating an advanced IPF progression.

A STE-derived impairment of RV-GLS and LV-GLS in an IPF patient at an early disease stage might suggest the introduction of cardioprotective drugs, particularly beta-blockers and statins. In this regard, data in the literature indicate that, despite the increased prevalence of cardiovascular risk factors among IPF patients, beta-blockers and statins are underprescribed in these patients in comparison to the general population [40]. The underprescription of beta-blockers and statins in IPF patients may be related to a decreased primary cardiovascular prevention, a possible result of poor medical attention to routine cardiovascular care. Moreover, blood pressure control is not always optimal in IPF patients, as highlighted by the results of this meta-analysis; accordingly, an accurate up-titration of antihypertensive therapy may be required in these patients.

Given the mild attenuation of RV-GLS and LV-GLS magnitude detected in IPF patients and the wide overlap of SMDs between IPF patients and controls, it is also important to consider that subtle changes in biventricular mechanics are not always related to intrinsic myocardial dysfunction. Indeed, it is noteworthy that a subclinical RV dysfunction may also be correlated to various degrees of anterior chest wall deformity and technical factors, such as tachycardia and/or suboptimal acoustic windows [41]. Therefore, in the clinical decision-making process, the physiological meaning of RV and/or LV-GLS attenuation in each single IPF patient should always be put in relation to the clinical picture and integrated with clinical, laboratory, and spirometric parameters and/or pulmonary hemodynamics.

### 4.4. Limitations of the Included Studies

The main limitations of the included studies were the small number of IPF patients examined in each study and their monocentric nature. Moreover, the fact that the majority of the included studies were performed in Italy (83.3% of total) may limit the generalizability of results. However, the present systematic review analyzed all the literature data currently available on IPF patients with mild-to-moderate stage of the disease, who had undergone a conventional 2D-TTE implemented with STE analysis of biventricular mechanics. In addition, the included studies were primarily focused on the echocardiographic assessment of both conventional and innovative indices of biventricular systolic function in IPF patients with mild-to-moderate lung disease, while information concerning IPF comorbidities and the current medical treatment were scanty. For this reason, we could not adequately evaluate the impact of relevant comorbidities and anti-fibrotic therapy on RV-GLS. Finally, it is noteworthy that STE use in clinical practice is limited. Indeed, this innovative methodology is not available in all centers and suffers from a number of limitations, such as intervendor variability and dependence on frame rate, on loading conditions, and on chest wall conformation [42,43,44,45].

## 5. Conclusions

Comprehensive STE examination of biventricular mechanics may allow clinicians to obtain incremental diagnostic information over conventional TTE in patients with early-stage IPF.

RV-GLS impairment is an early marker of subclinical myocardial dysfunction in mild-to-moderate IPF.

STE should be considered for implementation in clinical practice to early detect RV dysfunction in IPF patients without advanced lung disease.

## Figures and Tables

**Table 1:** Clinical characteristics and main findings of included studies [18,19,20,21,22,23].

Study Name, Country and Publication Year	Population	Mean Age of IPF Pts (% Males)	Study Design	Imaging Method	Main Findings in IPF Patients vs. Healthy Controls
D’Andrea A., et al. (2016) [18], Italy	IPF = 52 Controls = 45	66.7 (48.1)	Prospective	2D-TTE, STE	↔LV internal dimensions, LVMi, E/A, E/e’, LVEF; ↓LV-GLS; ↔TAPSE, TDI RV peak S’; ↑RV basal diameter, TRV, sPAP; ↓RV-GLS
D’Andrea A., et al. (2019) [19], Italy	IPF = 50 Controls = 50	61.2 (70)	Prospective	2D-TTE, ESE, STE	Resting findings: ↑RV basal diameter, sPAP; ↔E/e’, TAPSE, LV-GLS; ↓RV-GLS; Peak exercise findings: ↓Watt achieved, maximal HR, SpO2; ↑ sPAP; ↓RV-GLS
Buonauro A., et al. (2020) [20], Italy	IPF = 33 Controls = 30	70.1 (81.8)	Prospective	2D-TTE, 3D-TTE, STE	↔LVMi, LVEF; ↓transmitral E/A; ↑E/e’; ↓LV-GLS; ↑RV basal diameter, sPAP; ↓TAPSE, tricuspid E/A, RV-GLS, 3D-RVEF
Sonaglioni A., et al. (2020) [21], Italy	IPF = 50 Controls = 30	73.8 (72)	Retrospective	2D-TTE, STE	↔LVMi, LVEF, E/A, RV basal diameter, TAPSE; ↑E/e’, TRV, sPAP; ↓LASr, LV-GLS; ↔RASr, RV-GLS
Cobra S.B., et al. (2021) [22], Brazil	IPF = 20 Controls = 10	72.3 (50)	Prospective	2D-TTE, CPET, STE	Resting findings: ↑LAVi, ↔E/e’, ↓RV-FAC, TAPSE, ↑mPAP, sPAP, ↔RV-GLS; Peak exercise findings: ↑mPAP, ↓RV-GLS, ↓peak V’O2, O2 pulse, maximal HR
Santoro C., et al. (2022) [23], Italy	IPF = 50 Controls = 30	67 (74)	Retrospective	2D-TTE, STE	↔LV internal dimensions, LVMi, E/e’, LVEF, LV-GLS; ↓E/A; ↑RV basal diameter, TRV, sPAP; ↔TAPSE; ↓RV-GLS, TAPSE/sPAP, RV-GLS/sPAP

2D, two-dimensional; 3D, three-dimensional; CPET, cardiopulmonary exercise testing; FAC, fractional area change; GLS, global longitudinal strain; HR, heart rate; IPF, idiopathic pulmonary fibrosis; LASr, left atrial reservoir strain; LAVi, left atrial volume index; LV, left ventricular; LVEF, left ventricular ejection fraction; LVMi, left ventricular mass index; mPAP, mean pulmonary artery pressure; peak V’O2, peak oxygen consumption; RASr, right atrial reservoir strain; RV, right ventricular; RVEF, right ventricular ejection fraction; SpO2, peripheral arterial oxygen saturation; sPAP, systolic pulmonary artery pressure; STE, speckle tracking echocardiography; TAPSE, tricuspid annular plane systolic excursion; TDI, tissue Doppler imaging; TRV, tricuspid regurgitation velocity; TTE, transthoracic echocardiography. The symbol ↓ indicates a significant lower value of each variable in IPF Patients vs. Healthy Controls (*p*-value < 0.05); the symbol ↑ indicates a significant higher value of each variable in IPF Patients vs. Healthy Controls (*p*-value < 0.05); the symbol ↔ indicates the absence of statistical significant difference in the variable magnitude between IPF Patients and Healthy Controls (*p*-value > 0.05).

**Table 2:** Main clinical, respiratory, and echocardiographic parameters collected in IPF patients and controls by the included studies.

	IPF Patients	Controls	*p* Value	Number of Studies for Parameters Assessed (%)
Demographics				
Age (yrs)	68.5 (61.2–73.8)	66.4 (59.4–71.1)	<0.05	6 (100)
Male sex (%)	66.0 (48.1–74)	59.8 (40–70)	<0.05	6 (100)
Ethnicity				
European (%)	83.3	83.3	NS	6 (100)
Latin American (%)	16.7	16.7	NS	6 (100)
Anthropometrics				
BMI (Kg/m^2^)	27.5 (24.7–28.9)	26.7 (25.2–29.6)	<0.05	6 (100)
Cardiovascular risk factors and cardiovascular disease burden				
Current/ex smokers (%)	56.6 (31–75)	52.6 (28–66.7)	<0.05	6 (100)
Hypertension (%)	50.7 (44–60)	33.5 (17–50)	<0.05	3 (50)
Type 2 diabetes (%)	17 (12–20)	11.7 (0–23.4)	<0.05	4 (66.7)
History of CAD (%)	14 (14–14)	/	/	2 (33.3)
Respiratory parameters				
Room air SaO2 (%)	88.3 (83.4–93.2)	93.3 (92.5–94.1)	<0.05	2 (33.3)
PaO2 (mmHg)	75.4 (71.6–80.7)	/	/	2 (33.3)
FEV1 % predicted	75.5 (70.9–81.3)	/	/	4 (66.7)
FVC % predicted	68.7 (61–75.9)	/	/	6 (100)
FEV1/FVC (%)	84.3 (80.1–87)	/	/	3 (50)
DLCO % predicted	45.1 (35.8–53.9)	/	/	6 (100)
6MW distance (m)	419.4 (346.4–490)	/	/	5 (83.3)
Hemodynamics				
HR (bpm)	76.2 (71.9–79.5)	72 (68.2–77.6)	<0.05	4 (66.7)
SBP (mmHg)	135.6 (128.3–139)	129.3 (124–134.6)	<0.05	4 (66.7)
DBP (mmHg)	80.7 (78.9–82)	78.8 (76.5–80.5)	<0.05	4 (66.7)
Conventional echoDoppler variables				
IVS thickness (mm)	9.3 (9.1–9.5)	9.4 (9–9.8)	NS	2 (33.3)
PW thickness (mm)	8.5 (8.5–8.6)	8.6 (8.5–8.7)	NS	2 (33.3)
LV-EDD (mm)	48 (47.9–48.1)	48.1 (48–48.2)	NS	2 (33.3)
RWT	0.39 (0.36–0.44)	0.38 (0.36–0.42)	NS	4 (66.7)
LVMi (g/m^2^)	83.7 (73.2–95.3)	80.4 (75.7–91.7)	NS	4 (66.7)
LVEF (%)	60.5 (55.8–63)	61.4 (57.4–63.2)	NS	4 (66.7)
LAVi (mL/m^2^)	26.7 (20.8–33.9)	24.1 (14.7–32)	NS	3 (50)
E/A ratio	0.79 (0.71–0.92)	0.89 (0.75–0.97)	<0.05	4 (66.7)
E/e’ ratio	9.08 (5.3–14.4)	7.05 (4.3–9.6)	<0.05	6 (100)
RAVi (mL/m^2^)	15.5 (9.7–21.3)	12.5 (6.9–18.1)	NS	1 (16.7)
RV basal diameter (mm)	36.4 (32.5–39.8)	32.3 (28–35.9)	<0.05	5 (83.3)
TDI RV peak systolic velocity S’ (cm/s)	12.6 (11.5–13.9)	12.7 (12–13)	NS	4 (66.7)
TAPSE (mm)	21.1 (20.3–22)	22.4 (21–23.4)	<0.05	6 (100)
TRV (m/s)	2.66 (1.84–3.21)	2.17 (1.61–2.47)	<0.05	6 (100)
sPAP (mmHg)	34.6 (18.6–46.1)	24.3 (15.3–30.1)	<0.05	6 (100)
TAPSE/sPAP ratio (mm/mmHg)	0.65 (0.48–1.09)	0.96 (0.75–1.37)	<0.05	1 (16.7)
Myocardial strain parameters (%)				
RV-GLS (%)	18.6 (13.4–22.6)	22 (18.4–24.2)	<0.05	6 (100)
RV-GLS/sPAP ratio (%/mmHg)	0.55 (0.47–0.67)	0.92 (0.77–1.07)	<0.05	1 (16.7)
LV-GLS (%)	18.8 (15.9–20.9)	20.8 (18.8–22.7)	<0.05	5 (83.3)
LASr (%)	23.7 (18.8–28.6)	37.1 (31.9–42.3)	<0.05	1 (16.7)
Anti-fibrotic treatment (%)	42 (0–84)	/	/	2 (33.3)

Data are expressed as median and IQR. Significant *p*-values are in bold. 6MW, six-minute walking; BMI, body mass index; DBP, diastolic blood pressure; DLCO, diffusing capacity of the lungs for carbon monoxide; EDD, end-diastolic diameter; FEV1, Forced Expiratory Volume in the 1st second; FVC, forced vital capacity; GLS, global longitudinal strain; HR, heart rate; IPF, idiopathic pulmonary fibrosis; IQR, interquartile range; IVS, interventricular septum; LASr, left atrial reservoir strain; LAVi, left atrial volume index; LV, left ventricular; LVEF, left ventricular ejection fraction; LVMi, left ventricular mass index; NS, not significant. PaO2, arterial oxygen partial pressure; PW, posterior wall; RAVi, right atrial volume index; RV, right ventricular; RWT, relative wall thickness; SBP, systolic blood pressure; sPAP, systolic pulmonary artery pressure; SpO2, peripheral arterial oxygen saturation; TAPSE, tricuspid annular plane systolic excursion; TDI, tissue Doppler imaging; TRV, tricuspid regurgitation velocity.

**Table 3:** Results of meta-regression analysis of IPF effect on RV-GLS. GLS, global longitudinal strain; IPF, idiopathic pulmonary fibrosis; RV, right ventricular.

Moderators	Coefficient	Standard Error	95%CI Lower	95%CI Upper	*p*-Value
Age	0.10	0.12	−0.13	0.33	0.39
Hypertension	−0.12	0.11	−0.34	0.09	0.26
Type 2 diabetes	−0.09	0.18	−0.44	0.27	0.64
Smoking	−0.02	0.04	−0.09	0.06	0.67

## Data Availability

Data extracted from included studies will be publicly available on Zenodo (https://zenodo.org), pending acceptance by the journal.
